# Examining the reach and exposure of a mobile phone-based training programme for frontline health workers (ASHAs) in 13 states across India

**DOI:** 10.1136/bmjgh-2021-005299

**Published:** 2021-08-24

**Authors:** Jean Juste Harrisson Bashingwa, Neha Shah, Diwakar Mohan, Kerry Scott, Sara Chamberlain, Nicola Mulder, Sai Rahul, Salil Arora, Arpita Chakraborty, Osama Ummer, Rajani Ved, Amnesty Elizabeth LeFevre, Kilkari Impact Evaluation team, Smisha Agarwal

**Affiliations:** 1 Computational Biology Division, Department of Integrative Biomedical Sciences, Institute of Infectious Disease and Molecular Medicine (IDM), Faculty of Health Sciences, Cape Town, Western Cape, South Africa; 2 Johns Hopkins University Bloomberg School of Public Health, Baltimore, Maryland, USA; 3 Department of International Health, Johns Hopkins Bloomberg School of Public Health, Baltimore, Maryland, USA; 4 BBC Media Action, India, New Delhi, Delhi, India; 5 Computational Biology Division, Department of Integrative Biomedical Sciences, Institute of Infectious Disease and Molecular Medicine (IDM), Faculty of Health Sciences, Cape Town, South Africa; 6 BeeHyv Software Solutions Pvt. Ltd, Hyderabad, Telangana, India; 7 Oxford Policy Management, New Delhi, Delhi, India; 8 National Health Systems Resource Centre, New Delhi, Delhi, India; 9 International Health, Baltimore, Maryland, USA; 10 School of Public Health and Family Medicine, University of Cape Town, Cape Town, South Africa

**Keywords:** health systems, health systems evaluation

## Abstract

Mobile phones are increasingly used to facilitate in-service training for frontline health workers (FLHWs). Mobile learning (mLearning) programmes have the potential to provide FLHWs with high quality, inexpensive, standardised learning at scale, and at the time and location of their choosing. However, further research is needed into FLHW engagement with mLearning content at scale, a factor which could influence knowledge and service delivery. Mobile Academy is an interactive voice response training course for FLHWs in India, which aims to improve interpersonal communication skills and refresh knowledge of preventative reproductive, maternal, neonatal and child health. FLHWs dial in to an audio course consisting of 11 chapters, each with a 4-question true/false quiz, resulting in a cumulative pass/fail score. In this paper, we analyse call data records from the national version of Mobile Academy to explore coverage, user engagement and completion. Over 158 596 Accredited Social Health Activists (ASHAs) initiated the national version, while 111 994 initiated the course on state-based platforms. Together, this represents 41% of the estimated total number of ASHAs registered in the government database across 13 states. Of those who initiated the national version, 81% completed it; and of those, over 99% passed. The initiation and completion rates varied by state, with Rajasthan having the highest initiation rate. Many ASHAs made multiple calls in the afternoons and evenings but called in for longer durations earlier in the day. Findings from this analysis provide important insights into the differential reach and uptake of the programme across states.

**Summary box**

- Mobile Academy is a mobile phone-based refresher course for frontline health workers (FLHWs) in India.
- This paper focuses on the engagement of Accredited Social Health Activists (ASHAs), a specific cadre of FLHWs, during the government led scale up of Mobile Academy across 13 states in India.
- An estimated 41% of ASHAs listed in government databases initiated the course; 158 596 in the national version, while 111 994 initiated the course on state-based platforms.
- Initiation of the national version of Mobile Academy varied widely by state: Rajasthan had the highest number of ASHAs initiating, with 64% (37 078 ASHAs) of the state’s 57 567 ASHAs in the database initiating while West Bengal had the lowest initiation rate at just 1% (327 ASHAs of 51 068 total in the database).
- ASHAs spent an average of 5 hours over 10 calls to complete the course for the first time (some completed the course more than once).
- ASHAs called more times in the latter half of the day, but longer calls on average occurred earlier in the day.
- 15% of ASHAs spent between 240 and 260 min to complete the course and obtained a perfect or near-perfect score (43 or 44 out of 44).

## Background

Digital tools are increasingly being used by frontline health workers (FLHWs) in low/middle-income countries to support data capture, decision support, access to health information and in-service training. Training programmes—referred to as mobile learning (mLearning) programmes—have the potential to train large numbers of providers on standardised content, at low cost, and at the time and location of their choosing, without disrupting routine service delivery—refreshing learning provided via face-to-face training programmes and filling knowledge gaps.

Evidence on the effectiveness of mLearning programmes is emerging, with a number of studies having examined the impact of using mobile phone-based training or refresher courses in Senegal, South Africa, Iran, China, Ghana, Malawi and Tanzania; all with largely positive results.^1–8^ Evidence on the perceptions of mLearning programmes for health workers which use mobile phones, including apps and personal digital assistants, broadly suggest that they are complicated by a variety of contextual factors,^9^ but overall the programmes are associated with equivalent or sometimes even higher levels of knowledge^10^ as compared with traditional face-to-face learning approaches. Studies examining other technologies such as tablet-based or computer-based methods have similarly shown improvements in knowledge and quality of care.^11–17^ Despite the immense potential of mLearning programmes, evidence on available deployments to date is largely constrained to small scale pilots^18^ and focused on impact, with limited reporting on programmatic reach or user engagement.

India has the largest number of digitally enabled FLHWs globally.^19^ Accredited Social Health Activists (ASHAs) are a cadre of female FLHWs based in villages, trained to act as a bridge between the community and the health system. ASHAs counsel families on health issues, encourage service uptake through escorting women and children to receive health services, promote community mobilisation and provide basic first-aid services.^20^

Mobile Academy^21 22^ is an interactive voice response (IVR) mLearning programme for ASHAs which was developed in 2012 by BBC Media Action and scaled with the National Health Mission in three states (Bihar, Odisha and Uttar Pradesh) before it was adopted by the Ministry of Health and Family Welfare (MoHFW) in 2014. A new national version of the training course was then co-created by BBC Media Action with MoHFW, before it began to roll out in November 2015. The national version of Mobile Academy aims to refresh initial ASHA training and includes 11 chapters and 240 min of pre-recorded audio lessons covering topics on reproductive, maternal and child health, family planning and hygiene (online supplemental figure 1). ASHAs call into the service at a time that is convenient for them to listen to the audio lessons. At the end of each chapter, they complete a 4-question true/false self-assessment quiz intended to prompt continued engagement, and receive an accumulative pass or fail score at the end of the course. The course can be completed over multiple dial-ins; ASHAs can hang up at any point and then call back later to continue working through the content. Bookmarking technology linked to their mobile number returns to the user to the lesson they reached before hanging up previously. ASHAs who correctly answer half of the assessment questions pass the course and are eligible to receive a certificate of course completion. To date, Mobile Academy has scaled across 13 of India’s 36 states and territories and is estimated to have reached over 171 451 ASHAs.

10.1136/bmjgh-2021-005299.supp1 Supplementary data

**Figure 1:**
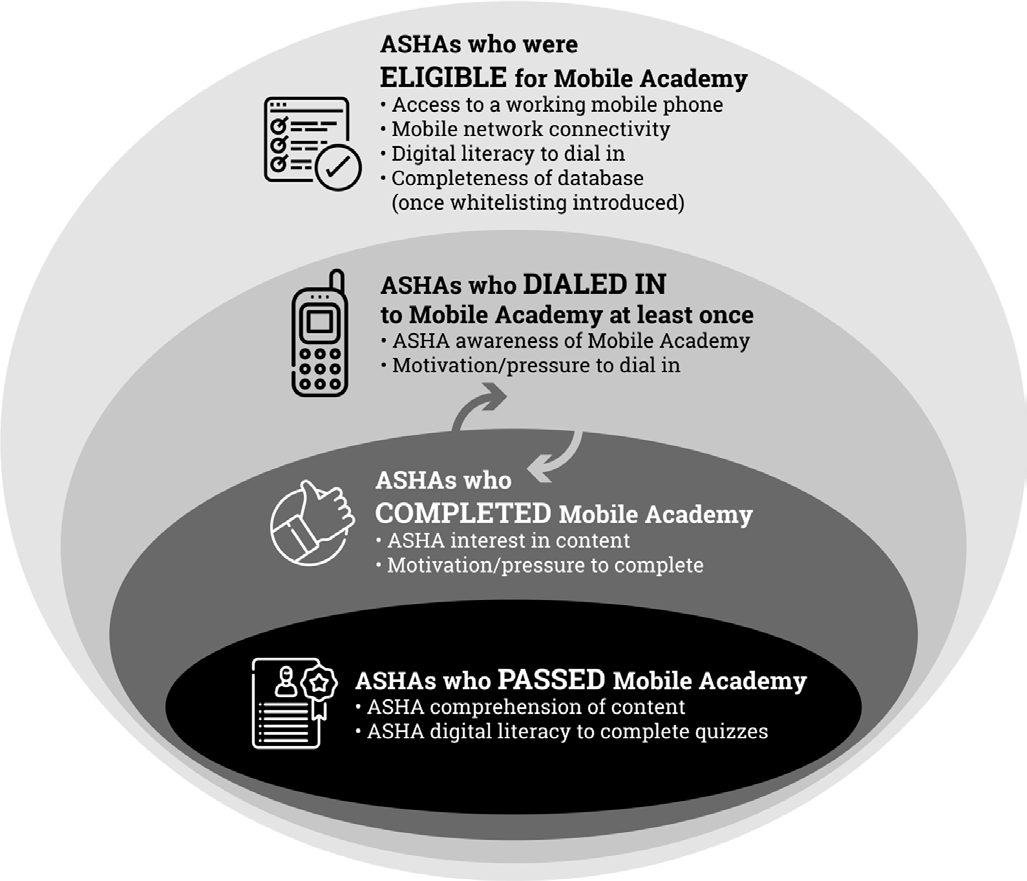
Conceptual framework of Mobile Academy access, uptake and engagement. ASHA, Accredited Social Health Activist.

MLearning offers much potential as a low cost, accessible and convenient supplement to face-to-face refresher training for healthcare providers operating on the frontlines of the health system. While qualitative research may offer important insights^23^ into its reported acceptability and broader perceptions about its utility, in this manuscript, we conceptualise the reach and engagement of the national version of Mobile Academy through examining the number of ASHAs who were eligible to dial in, the number of ASHAs who initiated the course, the number who completed it and the number who passed (figure 1 and online supplemental table 2). Using call data records from the national platform from 2015 to 2019 across 13 states, we first present data on Mobile Academy reach by assessing the proportion of ASHAs who initiated the national version of Mobile Academy. We next assess their engagement with Mobile Academy by considering the number of repeated completions as well as the time at which they called in. To assess ASHA performance on assessment modules, we examined the percentage of ASHAs who proceeded through all 11 chapters of the course and answered the 44 quiz questions. Lastly, we analysed time to completion and determinants of completion. This analysis does not include the 55 765 of ASHAs who completed Mobile Academy on state-based platforms in Bihar, Uttar Pradesh and Odisha.

10.1136/bmjgh-2021-005299.supp4 Supplementary data

## Over 24% of the ASHAs in the 13 states where Mobile Academy was implemented initiated the national version of the course

ASHAs accessed Mobile Academy by dialling in to a toll-free number. In some states, there was a short initial period of time where any mobile number could dial in and access the programme. However, all states eventually implemented a ‘whitelisting’ access policy. This policy only allowed mobile numbers registered to ASHAs in the government databases (government databases include the mother and child tracking system and reproductive and child health databases which are integrated with the Mobile Academy database) to dial in. Mobile Academy received 86 198 dial-ins from non-Mobile Academy database mobile numbers before whitelisting was implemented. These dial-ins are therefore anonymous, although results from a phone survey with ASHAs in six states, conducted by BBC Media Action in 2016, indicates that the vast majority of anonymous callers are likely to have been ASHAs calling in from non-registered mobile numbers. Without counting these anonymous dial-ins, we found that 24% (158 596) of the 656 831 ASHAs whose phone numbers were registered in the national database accessed Mobile Academy. These numbers do not take into account the state-based platforms in Bihar, Uttar Pradesh and Odisha, where 111 994 ASHAs initiated the programme.^24^

**Figure 2:**
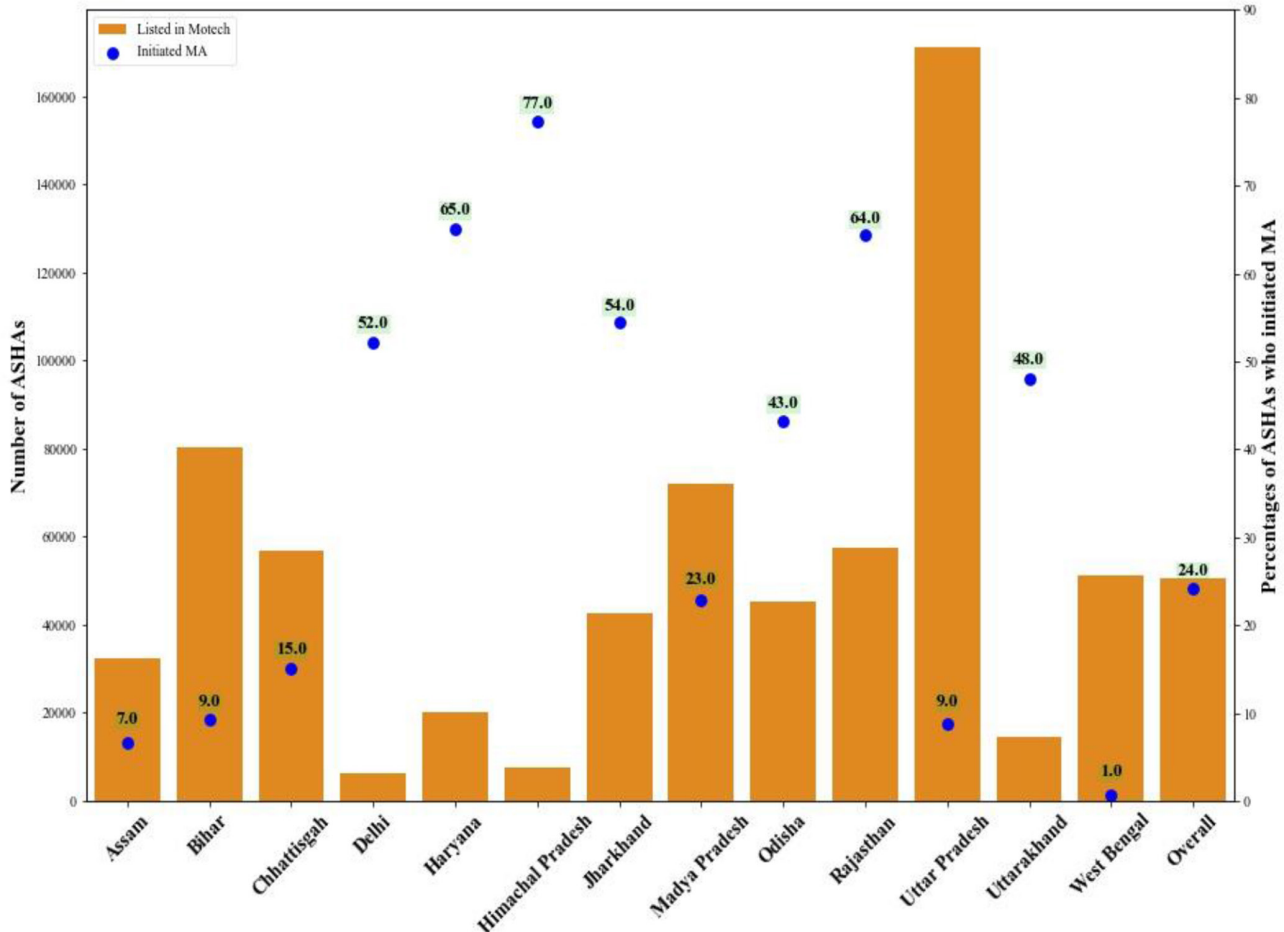
Initiation rate of the national version of MA of those ASHAs contained in the national MA database by state. ASHA, Accredited Social Health Activist; MA, Mobile Academy.

ASHA initiation of Mobile Academy varied by state over the years of implementation from 2015 to 2019 when Mobile Academy was transitioned to the government. Peak periods of initiation coincided with Mobile Academy rollout in many states, and were followed by subsequent peaks when state governments were either introducing or promoting the programme among their ASHAs. Uttar Pradesh had the highest number of ASHAs registered in the Mobile Academy database (170 998), but only a 9% initiation rate. Whereas, Delhi had less than 6128 ASHAs in the Mobile Academy database, but a 52% initiation rate. The overall 24% initiation rate on the national platform is largely driven by Rajasthan where 64% of ASHAs (37 078 out of the 57 567 ASHAs) in the Mobile Academy database initiated training (figure 2).

Initiation rates were driven primarily by four factors. First, the quality of underlying phone numbers entered into the Mobile Academy database (once whitelisting was implemented, which prevented ASHAs using alternative mobile numbers to access the course). Second, Mobile Academy promotion by state governments. Third, the monitoring and supervision of ASHAs’ take-up and progress through the course by ASHA supervisors. Lastly, the quality of mobile networks in areas where ASHAs live and work, including the quality of government issued SIM cards. An estimated 10% of non-whitelisted or anonymous ASHAs (8595) who took the course later registered their number in the Mobile Academy database. Information on the quality of data collected by state governments and imported into the MoHFW’s national databases not available, including the completeness or accuracy of phone numbers. Underlying limitations in data quality may explain some of variation in initiation rates observed across states.

## 81% of ASHAs who initiate Mobile Academy in the national version complete the programme

**Figure 3:**
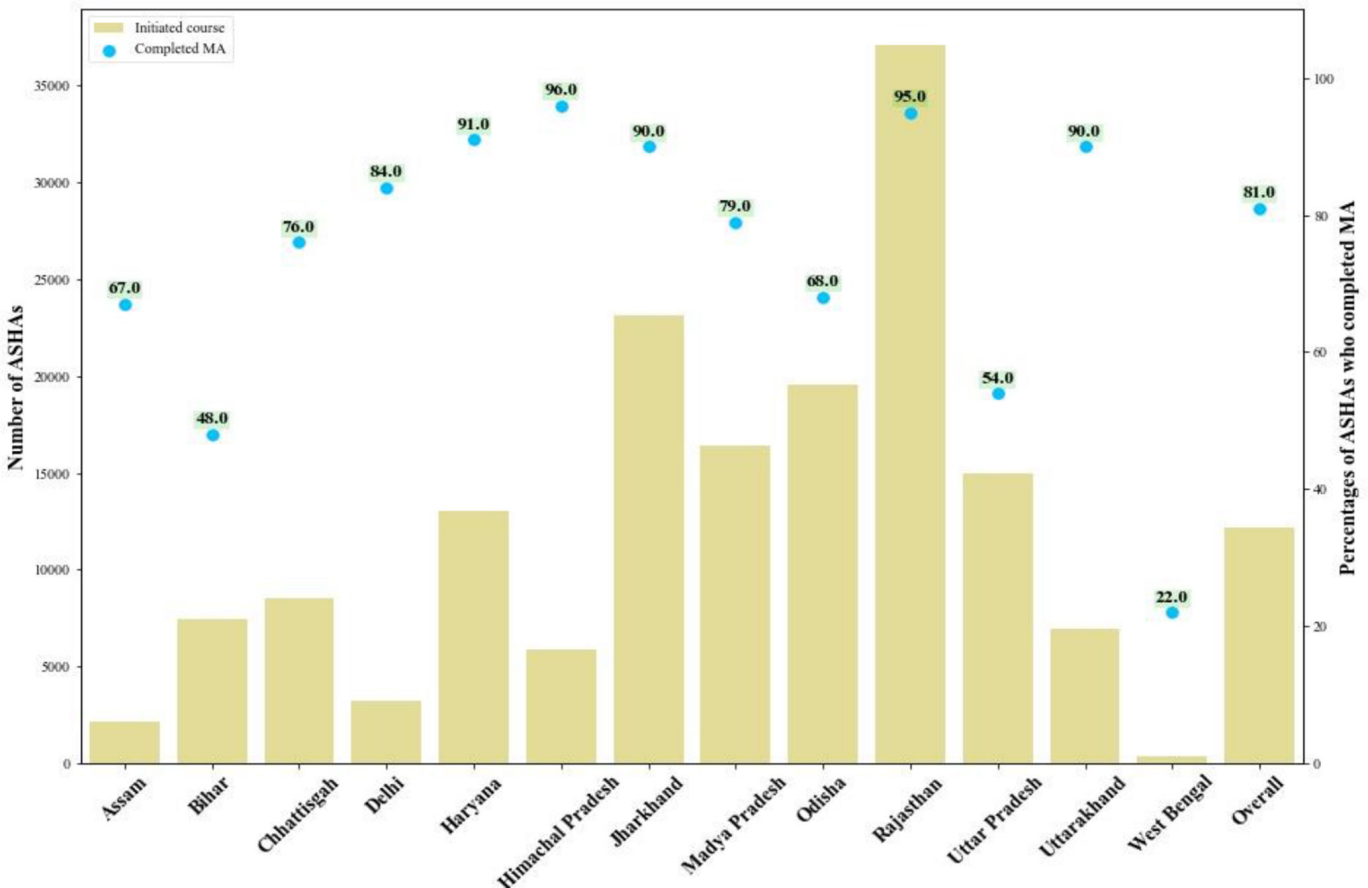
Completion rate among those ASHAs who initiated the national version of MA by state. ASHA, Accredited Social Health Activist; MA, Mobile Academy.

Mobile Academy completion rates for ASHAs who initiate the course were high, with an estimated 81% (128 135) of ASHAs who initiated the national version completing the course. Additionally, 11% of ASHAs who were initially anonymous users of Mobile Academy later registered their mobile numbers in the government’s databases to complete Mobile Academy. Mobile Academy may thus have acted an incentive for ASHAs to register their mobile numbers with the government. Across states, completion rates varied from 22% in West Bengal to 96% in Rajasthan. Rajasthan had the highest initiation and completion in terms of absolute numbers and percentages; a factor likely attributed to the Governments high level engagement with and promotion of the programme. Thus, Rajasthan drove both initiation (23% of total initiation population) and completion (27% of total completed) of the programme overall (figure 3).

## 75% of ASHAs who completed the national version of the course had a perfect or near-perfect score (43/44 or 44/44)

Following each of the 11 chapters, four yes/no questions were asked of ASHAs in an effort to prompt continued engagement with the course. However, an ASHA can ‘complete’ Mobile Academy without responding any of the questions posed at the end of each module, although she will not be eligible for a certificate if she does this. The course was designed this way because the human-centred design process that was used to create Mobile Academy identified that some ASHAs could not use IVR navigation, but were still able to benefit from the course just by listening to the content. Among the ASHAs who took the quizzes (the majority), those who chose to answer quiz questions and correctly answered 22 out of 44 were deemed to have ‘passed’ the course. From October 2015, details on ASHA performance on these questions were captured. Among those ASHAs who completed Mobile Academy, 99.7% (127 801) passed it, for example, correctly answered 22 or more of 44 quiz questions. The mean score was 42.5/44 and the median score was 44/44, with 75% of ASHAs earning a perfect score.

## Time to completion: over one-third of ASHAs completed the national version of Mobile Academy within 3 days of initiation

The 11 chapters and 44 questions which comprise Mobile Academy constitute 4 hours of content. ASHAs can proceed through the content once, or they can choose to repeat the lessons and chapters in the course as many times as they like to improve comprehension and recall. Once initiated, 36% of ASHAs completed the programme within 3 days; 12% within the first 24 hours. ASHAs spent an average of 5 hours over 10 calls for the first completion. Very few (1.9%) of ASHAs completed the course in one single long session, while one-third (30%) completed the course in more than 15 calls.

## Re-engagement: 17% of ASHAs took Mobile Academy more than once

In addition to allowing ASHAs to repeat lessons, chapters and quizzes to improve comprehension and recall, ASHAs were also allowed to repeat the course as many times as they liked to refresh their learning. Since January 2016 (when the database started tracking repeat attempts), 17% of ASHAs (21 225) completed the course more than once (table 1). When ASHAs chose to take the course a second time, they tended to proceed through the content more quickly: while 12% of ASHAs completed Mobile Academy in less than 24 hours their first time, 45% completed it within 24 hours their second time. Familiarity with the course content probably allowed the ASHAs to complete the course more quickly in later iterations of taking the course.

**Table 1:** Time gap between initiation and completion of Mobile Academy by completion iteration

Gap between first call and completion	First completion, n (%)	Second completion, n (%)	More completions, n (%)
<24 hours	15 278 (12)	6917 (45)	1137 (32)
1–3 days	30 421 (24)	1471 (10)	417 (12)
3–7 days	20 555 (16)	938 (6)	318 (9)
1–4 weeks	25 578 (20)	1667 (11)	464 (13)
>4 weeks	36 171 (28)	4233 (28)	1172 (33)

Whether they were completing Mobile Academy for the first time, or repeating the training, more than 50% completed it in 3 months or less and 75% completed it in under a year. These patterns indicate that the ASHAs used the flexible nature of the programme to fit their own schedule and called in when they had the time to do so. Fluctuations in network quality and phone access may also have influenced the time taken to complete the course. The completion time in days increased with subsequent completions. While we cannot know exactly why ASHAs repeated the course, they were encouraged to repeat it if they failed it, received a low score or just wanted to refresh their knowledge.

## Timing of engagement: ASHAs called more times in the latter half of the day, but longer calls on average occurred earlier in the day

**Figure 4:**
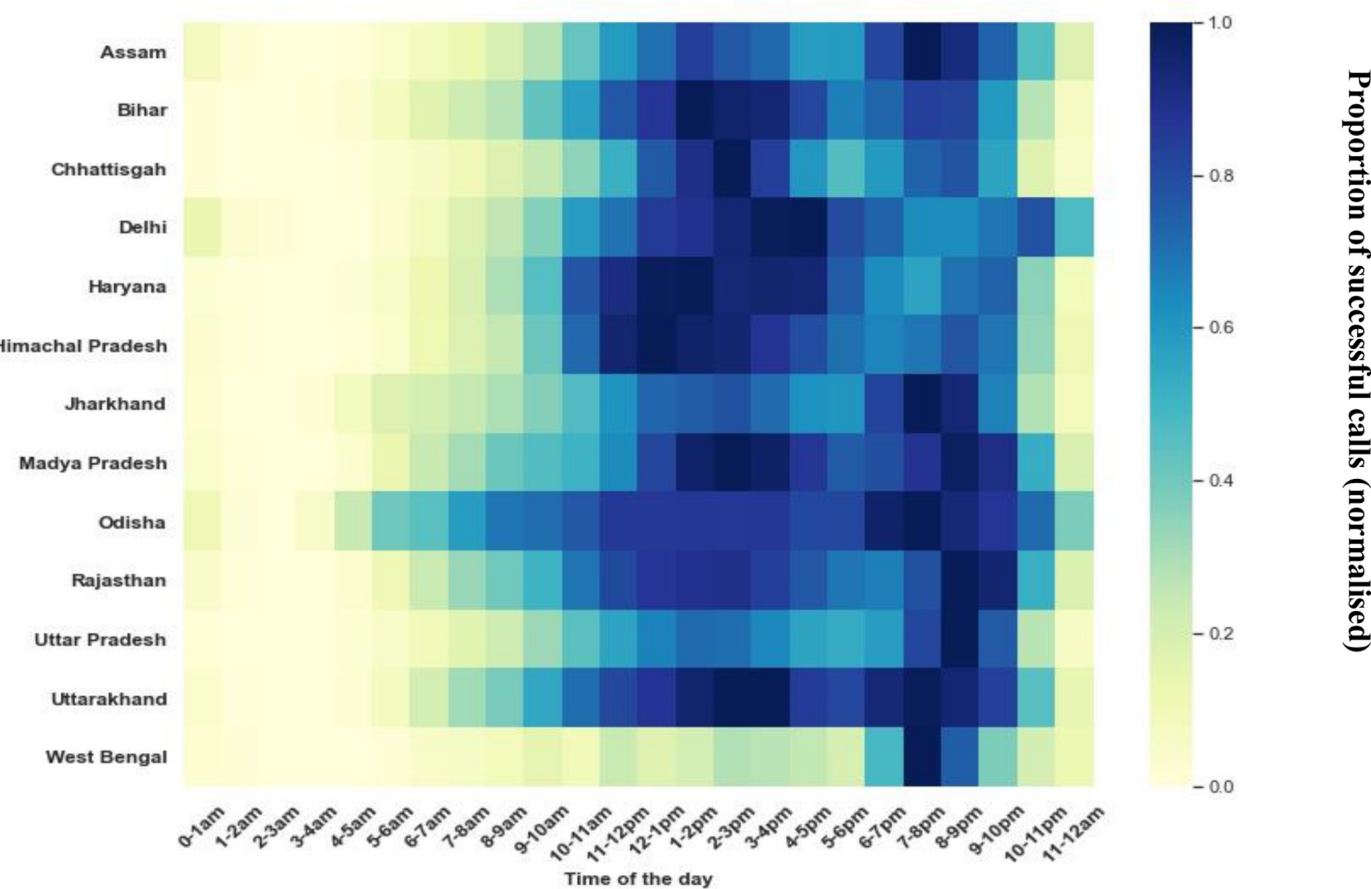
(A) Call volume across the day normalised* by state. (B) Mean duration (normalised) of successful calls by time of day and state.

To access ASHA engagement with the programme, trends in call volume and average call length throughout the day were assessed (figure 4A, B). Call volumes were greatest in the afternoon–evening time, with an average of 55% calls between 12:00 and 20:00, most likely when ASHAs were back in their homes and could spare the time to call into the programme for longer durations and call in more than once if their network connectivity was poor. Additionally, call drops (defined as a fewer than 3 min time gap between calls) were also frequent in the afternoon (54% from 12:00 to 20:00), indicating multiple calls could be going out due to poor network availability at this time. On the other hand, average call length throughout the day was much more diverse by state. The average call length was 22 min. Average call lengths varied from state to state with the highest (32 min) in Rajasthan and the lowest (16 min) in UP. Most states have the peak average call length around 03:00–05:00 except Haryana where the peak occurs between 13:00 and 14:00. Less than 3% of ASHAs call in between 01:00 and 05:00. This trend of long calls occurring in the early morning hours may be attributable to a few ASHAs working through the course as much as possible before their daily chores began or using ‘off peak’ hours when the mobile network was less burdened for longer duration calls.

## Determinants of completion: ASHAs who made multiple shorter calls and had longer gaps between calls were more likely to complete Mobile Academy

To examine factors that influence first time completion of Mobile Academy, we examined the gap between calls, the length of the call, the weekday and the time of the day when ASHAs called in (online supplemental table 1). The results suggest that ASHAs with a greater gap between calls had a higher likelihood of completion. The effect size increases for gaps between later calls as compared with the gap between earlier calls. For example, the greatest effect size occurs at gap of greater than 4 weeks between the fifth and sixth call with an increased OR of 3.87 (p<0.005) of completing Mobile Academy. This could be the result of deadlines set by state governments, where ASHA supervisors encouraged ASHAs to complete the course before the deadline. Additionally, length of the individual calls also influences completion: shorter calls are more likely to lead to completions rather than longer calls. This reinforces the importance of the bookmarking technology used which allowed users to return to the lesson they reached before hanging up previously vs starting anew. Weekday and time of the day for the call were not statistically significant determinants of the course completion.

10.1136/bmjgh-2021-005299.supp3 Supplementary data

## 21% of the ASHAs earned a near-perfect score on the national version of Mobile Academy’s assessment modules on their first attempt without repeating any content

Among ASHAs who completed Mobile Academy the first time and attained perfect (44/44) or near-perfect (43/44) scores, 21% completed the course in less than 240 min (cumulative) and 15% spent between 240 and 260 min. Overall, nearly two-thirds of ASHAs (72%) completed the course the first time with a score of 43 or 44.

## Summary learnings and implications for future programming

Mobile Academy was designed to refresh initial training received by ASHAs. It was not designed to introduce new content or enhance clinical skills. This cadre of FLHW is the largest of its kind globally, and once deployed ASHAs tend to remain in service for extended periods of time (Findings: cross-sectional surveys among ASHAs in Madhya Pradesh found that 60% of ASHAs had been in service for 4–10 years, 27% for 10+ years and 13% for <4 years.^25^). Given the volume of providers, and the length of time they remain in-service, low-cost alternatives to in-person training strategies are needed. Findings from this analysis suggest that Mobile Academy may be one such model for mLearning which can be used to refresh initial in-person training. Further research is needed to determine whether this modality might be appropriate for introducing new content to ASHAs.

Overall findings suggest that 24% of ASHAs contained in the National version of the Mobile Academy database initiated the training course. A further 50% of the 111 994 ASHAs completed Mobile Academy on state-based platforms in Bihar, Odisha and UP. Together, this represents 41% of the estimated total number of ASHAs registered in the government database across 13 states. To bolster programmatic reach, the data on ASHAs and their phone numbers need to be updated more frequently in government tracking registries (online supplemental figure 2). Among ASHAs that initiated Mobile Academy, the vast majority (81%) went on to complete it and almost all (97%) passed it. This suggests that ASHAs were sufficiently motivated (extrinsically and/or intrinsically) to proceed through the course—repeating lessons, chapters and quizzes as needed to increase comprehension and recall. Over 20% of all ASHAs who initiated Mobile Academy completed it with a perfect or near-perfect quiz scores on their first attempt, and without repeating any individual modules or quizzes. High ASHA performance on these quizzes may suggest that ASHAs had a strong recall of lessons learnt from the face-to-face training provided by the government. However, given that the purpose of the quizzes was to prompt continued engagement, they may not be appropriate for measuring comprehension or identifying knowledge gaps. Efforts to explore the determinants of Mobile Academy completion highlighted the importance of the bookmarking technology used which allowed ASHAs to complete the course in several sessions; returning each time to the place where they previously left off. ASHAs who made shorter call duration calls in to Mobile Academy and had longer gaps between calls were more likely to complete the course; suggesting that the capacity to consume smaller blocks of content over longer periods of time facilitates completion.

10.1136/bmjgh-2021-005299.supp2 Supplementary data

## Contextualising findings against other mLearning programmes

Mobile Academy is a unique example of a scaled direct to consumer strategy for training FLHWs. In Senegal, a similar approach of using IVR technology coupled with text messages was piloted in nurses (n=20) to provide 8 weeks of in-service training on contraceptive side effects and misconceptions.^4^ While the training was deemed acceptable to trainees and associated with knowledge gains,^4^ its scale of implementation is comparatively minute and as such, difficult to compare with Mobile Academy. Elsewhere mLearning programmes have used alternative strategies with regard to content delivery, modality, health focal areas and target providers. However, all are similarly constrained by the scale of implementation and lack of evidence on reach, exposure and impact. In Nigeria, tablets were used to enhance provider awareness about Ebola.^15^ In rural Guatemala, nurses’ mobile phones were used to support tele-care.^26^ In Malawi^13^ and Bangladesh,^27^ mobile phones have been used to foster digital skills development. Mobile phones have also been used to train midwives in the management of pre-eclampsia in Iran^28^ and support the management of viral infections in China.^2^ This collective body of mLearning programmes provides a general overview of potential use cases, while underscoring the need for more rigorous evaluation, particularly of solutions at scale. The absence of this evidence hampers decision making on the optimal design of mLearning programmes and recommendations for their appropriate use. It may well be that in circumstances where new skills are being introduced or its necessary to re-enforcement clinical skills, face to face or blended strategies, which offer partial face to face training with some measure of digitally enabled learning, yield greater impact despite their higher cost. However, for a cadre like ASHAs (of which there are nearly 1 million across India) concerned predominately with behaviour change communication, mLearning programmes like Mobile Academy are a low cost, feasible, rapid option for reinforcing initial trainings where face to face alternatives may be unaffordable. That said, they are not without their challenges. Contextual factors including digital literacy, mobile network stability, coupled with programme design decisions including modality of delivery, duration, health focus areas and embedded assessments, may influence uptake and impact on provider knowledge and service delivery.

## Conclusion

Mobile Academy is one of the largest mLearning programme for FLHWs in the world and succeeded in reaching an estimated 41% of ASHAs across state and national platforms. Ours is the first analysis of a mLearning programme for FLHWs at scale which aims to use system-generated data to glean insights on reach and beneficiary engagement. Differences in FLHW initiation across states provide insight into the differential rollout of the programme, as well as the broader quality of data in government tracking registries. Understanding how ASHAs engaged with the service—defined in terms of completion, time to completion and performance on quizzes—seeks to inform future design efforts. Future research should aim to understand barriers to initiation and link receipt of training to service delivery and health impacts.

## Data Availability

Available upon request.
