# Supplementary figures and images for: Examining the reach and exposure of a mobile phone-based training programme for frontline health workers (ASHAs) in 13 states across India
Source: BMJ Glob Health. 2021 Aug 24;6(Suppl 5):e005299. doi: 10.1136/bmjgh-2021-005299 (PMC8386225; doi:10.1136/bmjgh-2021-005299)

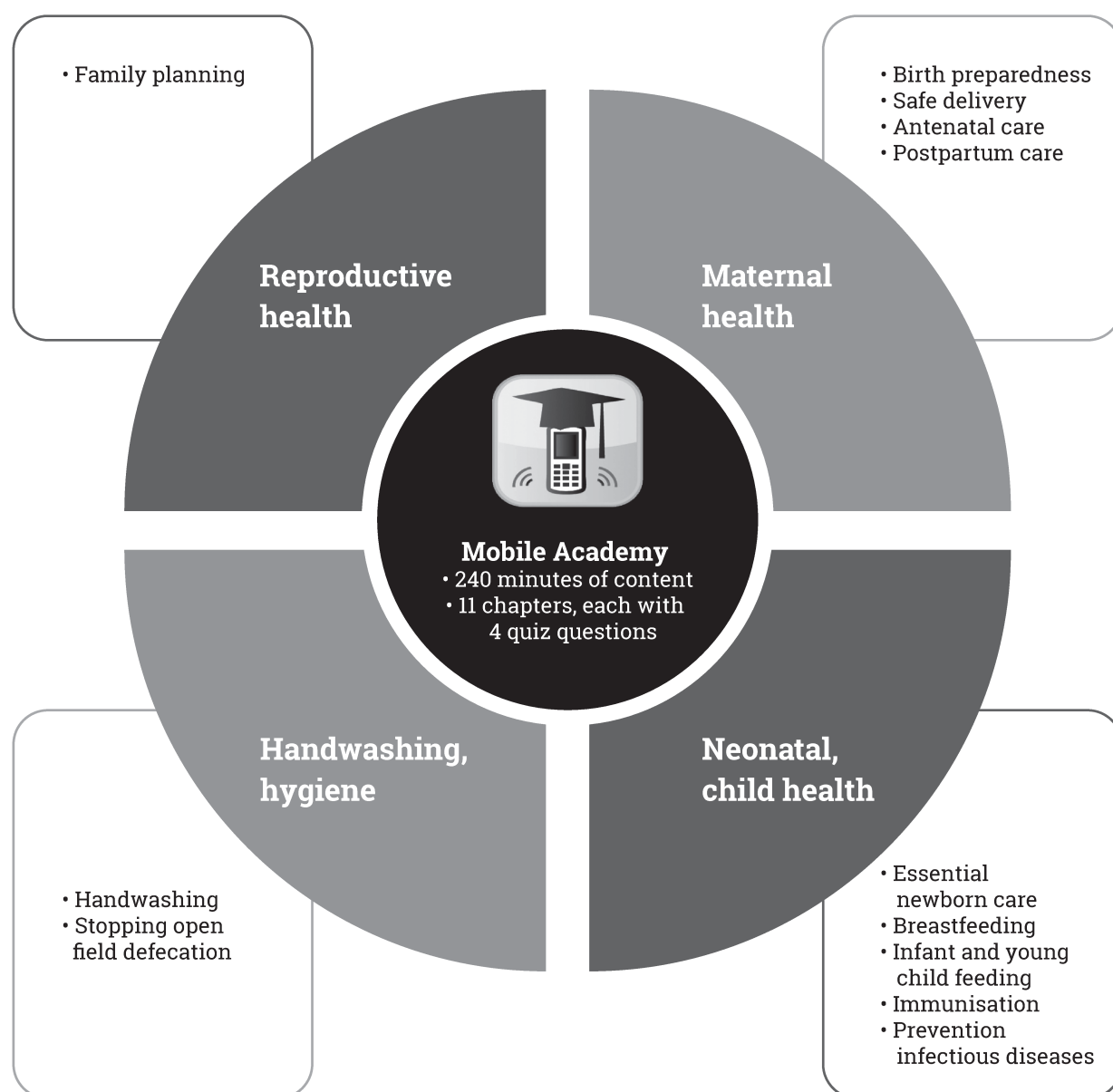

Supplement: Supplementary data [file bmjgh-2021-005299supp001.pdf]

**Supplementary Figure 1.** Active ASHA coverage per 1000 pregnant women in rural areas of 13 India states.

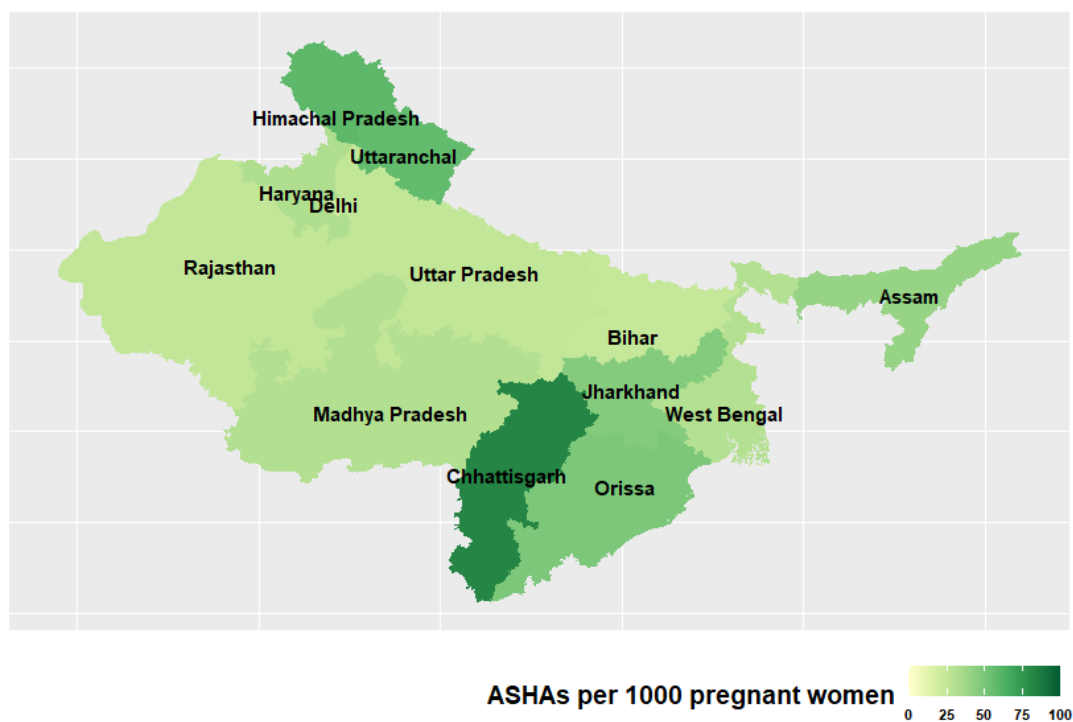

Supplement: Supplementary data [file bmjgh-2021-005299supp002.pdf]
