# Supplementary material for: Examining the reach and exposure of a mobile phone-based training programme for frontline health workers (ASHAs) in 13 states across India
Source: BMJ Glob Health. 2021 Aug 24;6(Suppl 5):e005299. doi: 10.1136/bmjgh-2021-005299 (PMC8386225; doi:10.1136/bmjgh-2021-005299)
Supplement: Supplementary data [file bmjgh-2021-005299supp004.pdf]

Supplementary Table 2. Mobile Academy initiation, completion, and pass rates by State

State Name	Total ASHAs	Initiated		Completed		Passed	
	N	n	%	n	%	n	%
Assam	32,300	2,125	7	1,420	4	1,386	4
Bihar	80,315	7,422	9	3,564	4	3,525	4
Chhattisgarh	56,677	8,536	15	6,518	12	6,453	11
Delhi	6,128	3,192	52	2,688	44	2,666	44
Haryana	20,057	13,053	65	11,851	59	11,771	59
Himachal Pradesh	7,573	5,861	77	5,652	75	5,579	74
Jharkhand	42,445	23,100	54	20,826	49	20,679	49
Madhya Pradesh	72,060	16,414	23	12,892	18	12,870	18
Odisha	45,173	19,547	43	13,259	29	13,062	29
Rajasthan	57,567	37,078	64	35,076	61	35,005	61
Uttar Pradesh	170,998	14,991	9	8,087	5	7,992	5
Uttarakhand	14,470	6,950	48	6,230	43	6,212	43
West Bengal	51,068	327	1	72	0	72	0
