# Supplementary material for: Examining the reach and exposure of a mobile phone-based training programme for frontline health workers (ASHAs) in 13 states across India
Source: BMJ Glob Health. 2021 Aug 24;6(Suppl 5):e005299. doi: 10.1136/bmjgh-2021-005299 (PMC8386225; doi:10.1136/bmjgh-2021-005299)
Supplement: Supplementary data [file bmjgh-2021-005299supp003.pdf]

Supplementary Table 1. Odds ratios of factors influencing Mobile Academy completion

	OR	LL	UL	p value	sig
**Gap between first and second call-in to MA**					
<24 hours	Ref				
1-3 days	1.02	0.96	1.09	0.56	
3-7 days	1.14	1.04	1.25	0.01	**
1-4 weeks	1.17	1.07	1.28	<0.001	***
>4 weeks	1.65	1.51	1.81	<0.001	***
**Gap between second and third call**					
<24 hours	Ref				
1-3 days	1.12	1.05	1.19	<0.001	***
3-7 days	1.25	1.13	1.38	<0.001	***
1-4 weeks	1.38	1.25	1.53	<0.001	***
>4 weeks	2.02	1.81	2.26	<0.001	***
**Gap between third and fourth call**					
<24 hours	Ref				
1-3 days	1.16	1.09	1.24	<0.001	***
3-7 days	1.44	1.30	1.59	<0.001	***
1-4 weeks	1.71	1.55	1.90	<0.001	***
>4 weeks	2.52	2.23	2.85	<0.001	***
**Gap between fourth and fifth call**					
<24 hours	Ref				
1-3 days	1.25	1.18	1.33	<0.001	***
3-7 days	1.85	1.67	2.04	<0.001	***
1-4 weeks	2.16	1.95	2.40	<0.001	***
>4 weeks	2.93	2.59	3.32	<0.001	***
**Gap between fifth and sixth call**					
<24 hours	Ref				
1-3 days	1.23	1.16	1.31	<0.001	***
3-7 days	1.78	1.61	1.97	<0.001	***
1-4 weeks	2.24	2.01	2.48	<0.001	***
>4 weeks	3.87	3.43	4.35	<0.001	***
**Length of first call**					
0-3 mins	Ref				
3-10 mins	1.11	1.04	1.18	<0.001	**
10-30 mins	1.03	0.96	1.10	0.42	
30-60 mins	0.85	0.78	0.92	<0.001	***
>60 mins	0.59	0.54	0.65	<0.001	***
**Length of second call**					

0-3 mins	Ref				
3-10 mins	0.97	0.91	1.04	0.38	
10-30 mins	0.91	0.85	0.97	<0.001	**
30-60 mins	0.67	0.62	0.73	<0.001	***
>60 mins	0.53	0.48	0.58	<0.001	***
**Length of third call**					
0-3 mins	Ref				
3-10 mins	0.91	0.85	0.98	0.01	*
10-30 mins	0.81	0.76	0.86	<0.001	***
30-60 mins	0.58	0.54	0.63	<0.001	***
>60 mins	0.44	0.40	0.49	<0.001	***
**Length of fourth call**					
0-3 mins	Ref				
3-10 mins	0.92	0.86	0.98	0.01	*
10-30 mins	0.80	0.75	0.85	<0.001	***
30-60 mins	0.58	0.53	0.63	<0.001	***
>60 mins	0.43	0.39	0.47	<0.001	***
**Length of fifth call**					
0-3 mins	Ref				
3-10 mins	0.86	0.80	0.92	<0.001	***
10-30 mins	0.73	0.69	0.78	<0.001	***
30-60 mins	0.49	0.45	0.53	<0.001	***
>60 mins	0.37	0.33	0.40	<0.001	***
**Weekday**					
Friday	Ref				
Monday	0.96	0.88	1.05	0.35	
Tuesday	1.05	0.96	1.14	0.29	
Wednesday	1.00	0.92	1.09	1.00	
Thursday	1.06	0.97	1.15	0.19	
Saturday	0.99	0.91	1.08	0.90	
Sunday	1.05	0.95	1.15	0.34	
